# Improvement of biochemical methods of polyP quantification

**DOI:** 10.15698/mic2017.01.551

**Published:** 2016-12-29

**Authors:** Samuel Bru, Javier Jiménez, David Canadell, Joaquín Ariño, Josep Clotet

**Affiliations:** 1 Department of Basic Sciences, Faculty of Medicine and Health Sciences. Universitat Internacional de Catalunya. Barcelona, Spain.; 2 Departament de Bioquímica i Biologia Molecular and Institut de Biotecnologia i Biomedicina, Universitat Autònoma de Barcelona. Cerdanyola del Vallès, Spain.

**Keywords:** polyphosphate, yeast, Saccharomyces cerevisiae, neutral-phenol

## Abstract

Polyphosphate (polyP) is an abundant and physiologically important biomolecule for virtually any living cell. Therefore, determination of changes in cellular content of polyP is crucial for its functional characterization. Determination of cellular polyP has been performed by many different methods, and the lack of a standardized procedure is possibly responsible for the large dispersion of results found in the relevant literature. For a relatively simple organism, such as the yeast *Saccharomyces cerevisiae*, this variation can be up to 12-fold. polyP extraction and determination of free phosphate released by enzymatic degradation of the polymer is a method quite common and relatively straightforward for polyP determination. By using the yeast *S. cerevisiae* as model, we have experimentally evaluated the different steps in this procedure in order to identify critical issues that might explain the disparate reported results. As the main output of this evaluation we propose a straightforward and robust procedure that can be used as gold standard protocol for cellular polyP purification and determination from unicellular organisms, thus providing consistency to measurements and facilitating inter-laboratory comparisons and biological interpretation of the results.

## INTRODUCTION

Inorganic polyphosphate (polyP) is a linear polymer of orthophosphate with many biological functions in both prokaryotic and eukaryotic organisms. In microorganisms, polyP plays an important function as stores of phosphorus and energy, in cation homeostasis, and in adaptation to stress conditions 1. Very recently, a role for polyP in post-translational modification of Lys residues in proteins has been reported 2. In complex organisms, as humans, this polymer is involved in many diverse functions such as blood coagulation, bone formation, immune response, regulation of calcium level in mitochondria, or neurotransmission (see 3 and references herein). In addition, polyP is used in diverse industrial processes, including water treatment, and as fertilizer in agriculture 4. Therefore, research on polyP is of great relevance in a variety of fields, ranging from the environmental to the biological sciences.

In the past, quantification of intracellular polyP content has been carried out by means of a diversity of approaches and methodologies, often yielding results that are difficult to compare. One of the most commonly employed methods to quantify polyP has been ^31^P NMR 5,6,7. While this technique has produced a foundational knowledge in the field of polyP research, it suffers of significant drawbacks. For instance, ^31^P NMR only detects phosphorus-containing molecules on the basis of bond class. Therefore, the method is not ideally suited to distinguish between polyP and molecules that also can contain phosphoanhydride bonds, such as nucleotides. Moreover, the abundant presence of P-ester compounds can obscure the comparatively smaller polyP signal in the majority of biological samples. In addition, usually NMR equipment is not well suited for time-resolved experiments in which environmental conditions must be quickly changed.

Staining with relatively specific dyes has been widely used for monitoring polyP accumulation both *in vivo* and *in vitro*. An example is the utilization of the metachromatic interaction of polyP with toluidine blue 8, although the most widely used method is based on the interaction between polyP and the fluorochrome 4′,6-diamidino-2-phenylindole (DAPI). DAPI is usually used for DNA detection, because blue fluorescence is apparent when the stained cells are viewed under UV light. However, the binding of DAPI to polyP also shifts the peak of DAPI and the fluorescence intensity at this shifted wavelength is proportional to the concentration of polyP 9,10. Although previous studies concluded that the presence of DNA do not complicate the fluorimetric quantification of polyP with DAPI, it has recently been described that RNA, inositol phosphates, amorphous calcium phosphates and nucleotides can cause significant interference at the wavelengths used to measure polyP, concluding that the DAPI-polyP interaction is sensitive to sample composition 7,11,12,13,14 and can lead to high variation between samples. Recently a new method for measuring polyP based on spectromicroscopy (Raman microscopy) has been developed 13,15. Raman microscopy is a fast evolving technology, but currently its sensitivity is relatively low and requires equipment not widely available.

Protocols that determine polyP concentration biochemically offer an appealing alternative to the methods discussed above, as exemplified by the early determination of polyP levels in different mammalian cells and tissues performed by the Kornberg's laboratory 16. Such enzymatic methods are based on the use of polyP kinase, followed by ATP measurement 17,18, or that of exopolyphosphatase followed by determination of free orthophosphate 19,20,21, and have been used in a variety of cells, from microorganisms to mammalian tissues. These methods are widely used because of their high sensitivity (particularly if radioactive phosphate is used), but they are not devoid of problems. For instance, they generally require of purification procedures, which can lead to erratic and/or low yields and, currently, no commercial sources exist for the required enzymes. In addition, even for enzyme-based protocols, the disparity of procedures used for purification and assays complicates inter-laboratory comparisons.

Possibly, the lack of a truly validated method to quantify cellular polyP content has been a drawback that has hampered the advance in the field. In fact, after evaluation of the relevant literature concerning the budding yeast *Saccharomyces cerevisiae*, we noticed a large disparity in the polyP content reported for this yeast that was difficult to explain on the basis of different genetic backgrounds or growth conditions. We considered that, at least in part, such inconsistency could derive from the use of different methods for polyP determination. In this work we analyze and compare possible alternatives for the main steps of exopolyphosphatase-based methods for polyP quantification, discuss the relative advantages, and propose a unified protocol for polyP determination that will help in the advance of the research of this polymer.

## RESULTS AND DISCUSSION

### Large discrepancies in determination of polyP cellular content claim for standardized determination methods

**Figure 1:**
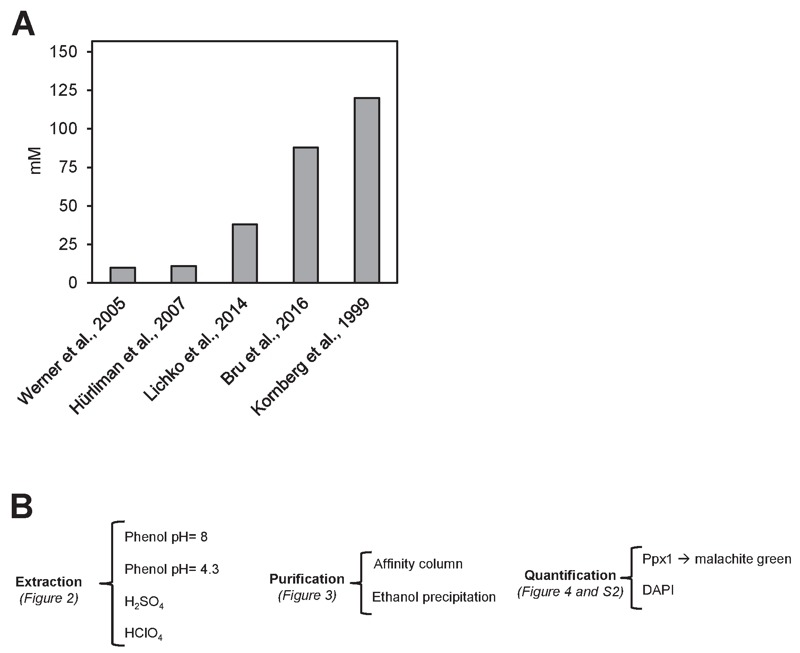
FIGURE 1: High variability on polyP determination in *S. cerevisiae.* **(A) **polyP content in mM concentration according to different authors. Values were converted to mM according to the following assumptions: i) The volume of the haploid cell is 42 fl, ii) One OD_660_ unit equals to 2.6 x 10^7^ cells and represents 50 µg of RNA. The Mw for the [-PO_3_^-^-] monomer is 79 g/mol. **(B) **Flow chart of this work, showing the different possibilities explored in each of the three parts needed for determining polyPs: extraction, purification and quantification.

A major issue when any cellular metabolite from a given source is determined is to evaluate whether the values obtained are congruent with those previously reported under similar circumstances (growth conditions, genetic background, etc.) in the literature. In the case of polyP content in yeast cells, this is a difficult task because, in many cases, the units employed are different (i.e. μg/OD unit, μmol/mg RNA, nmol Pi/10^n^ cells, and so on). We have made an attempt to integrate data from the literature in a common frame of measurement units (mM Pi). The result of this transformation applied to five independent reports 22,23,24,25,26 is shown in Figure 1. As it can be seen, there are large differences (up to 12-fold) in the amount of cellular polyP determined. It might be argued that this disparity could result from the use of different genetic backgrounds in the experiments. However, in several of the examples included in Figure 1, the BY4741 strain, or very close derivatives, were employed. The use of different backgrounds, even if it can result in some fluctuations, can hardly explain the large variations observed (our own unpublished data, see also reference 25). In addition, the Pi content in the media used and the growth phase of the cultures were fairly consistent among the different experiments. Therefore, it is reasonable to conclude that a substantial component of such variations must reside in the use of different protocols for polyP extraction, purification and quantification. In consequence, we considered necessary to carry out a comparative analysis of different options than can be proposed for each of these steps (Figure 1B).

### Influence of the extraction method on polyP quantification

**Figure 2:**
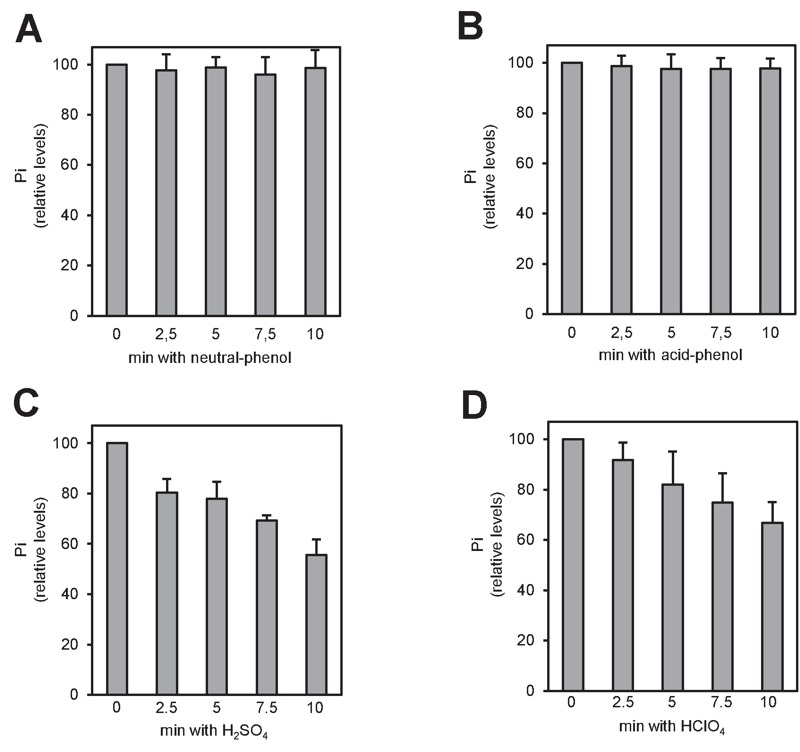
FIGURE 2: polyP is unstable when strong acids are employed during the extraction process. Relative amount of polyP after treatment with the different extraction solutions. Commercial polyP (4 μg) was incubated at different times with the different extraction solutions: **(A)** neutral-phenol at 4°C, **(B)** acid-phenol at 4°C, **(C)** 1 M H_2_SO_4_ at room temperature and **(D) **1 M HClO_4_ at 4°C. The mixes were neutralized, purified using affinity columns and the polyP eluted with MilliQ water. polyP amount was determined from the amount of Pi produced upon treatment with rPpx1. The graphs represent the percentage of polyP relative to time zero of each condition. Mean ± SEM from 3 independent experiments is shown.

There is plenty of evidence that polyP polymers are labile to acidic conditions 27. In fact, acid treatment at high temperature has been used for polyP quantification upon hydrolysis of the polymer 28. However, some methods for polyP extraction from cells or tissues involve the use of strong acids (usually sulfuric or perchloric). Therefore, we considered the possibility that this step might influence in the recovery of polyP from its original source. To evaluate this, we incubated 4 µg of commercial polyP for different periods of time with the reagents more frequently used in the literature: a phenol-based extraction solution at pH 8.0 (Figure 2A), the same solution at pH 4.8 (Figure 2B), 1 M sulfuric acid (Figure 2C) or 1 M perchloric acid (Figure 2D). As it can be observed, treatment with strong acids results in a time-dependent decrease in the amount of measured polyP, which was reduced up to one half of the untreated aliquot in as little as 10 min. This implies that some commonly used extraction methods would result in polyP degradation at this step, with the consequent impact in the final quantification.

### The purification method strongly influences the size and amount of recovered polyP

**Figure 3:**
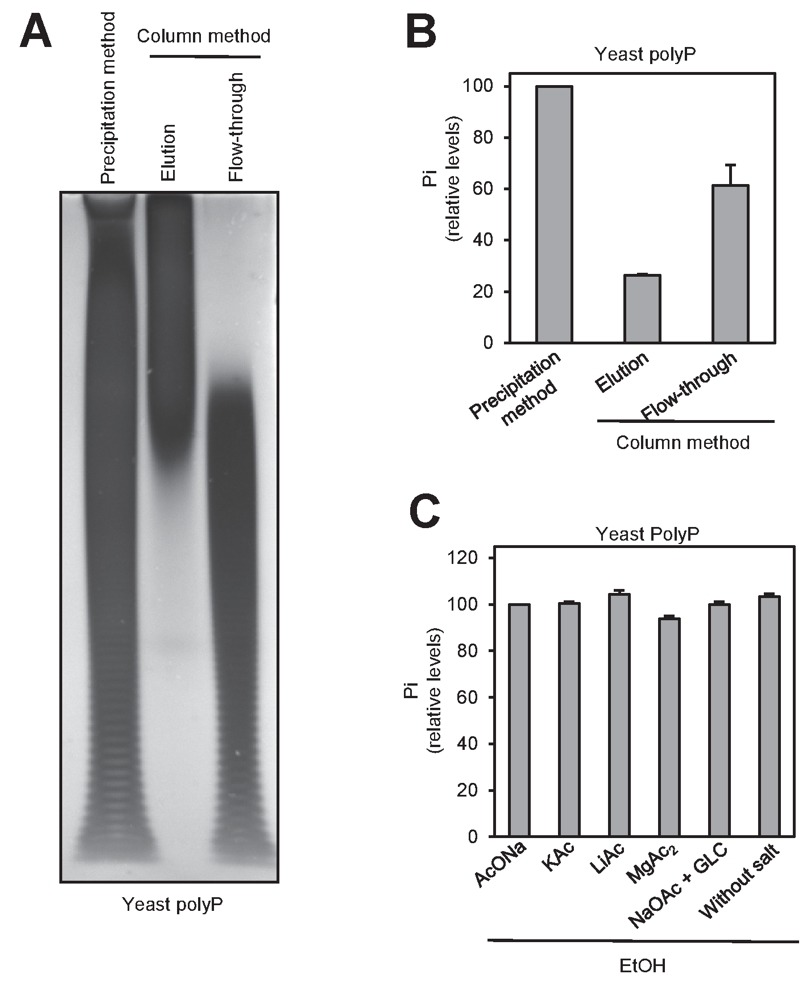
FIGURE 3: Ethanol precipitation method yields a broader spectrum of polyP sizes than affinity column purification. **(A)** PAGE and DAPI staining of polyP differently purified. polyP was extracted using the neutral phenol/chloroform procedure from a yeast pellet equivalent to 10^7 ^logarithmically growing yeast cells. The aqueous phase was treated with DNAse and RNAse solution, and purified by affinity columns or by ethanol precipitation. The resulting polyP fractions: precipitated (in the case of the ethanol) and eluted and flow-through (in the case of the affinity column) were analyzed by PAGE followed by DAPI staining. **(B)** Percentage of polyP relative to the purification method. polyP amount was determined using the same fractions obtained in panel A. The graph represents the Mean ± SEM from 3 independent experiments. **(C)** Relative amount of polyP obtained after the precipitation of polyP in the presence of different monovalent salts, divalent salts and a carrier. Aqueous phase from panel A was used. The graph represents the percentage of polyP relative to precipitation with NaOAc. Mean ± SEM from 3 independent experiments is shown. GLC, glycogen.

Two of the most frequently used methods for polyP purification upon extraction are affinity chromatography (usually silica-based) and ethanol precipitation. Reported evidence indicated that different extraction methods may result in size fractionation of recovered polyP 29. To test possible purification method-dependent differences in the length and recovery of polyP, we extracted polyP from two aliquots of yeast cultures by the phenol/chloroform (pH 8.0) method and samples were purified either by ethanol precipitation or by column chromatography (see Materials and Methods). Aliquots of the ethanol-precipitated material, as well as of the flow-through and the eluate of the chromatography were analyzed by polyacrylamide electrophoresis. As shown in Figure 3A, the chromatography step results in a dramatic size-dependent fractionation: polyP chains shorter than 60-80 residues were not retained by the column and appeared in the flow-through (which is usually discarded). This fractionation by size drastically decreased polyP recovery in the eluate which, upon quantification by degradation with rPpx1 and determination of the released phosphate, was estimated to be around 30-40% of the initial amount of polymer measured after the ethanol precipitation method (Figure 3B). Therefore, while extraction of polyP with the neutral phenol/chloroform method does not appear to introduce significant size selection bias (compare Figure 3A in this work with Figure 3 in reference 29), subsequent column purification results in size fractionation leading to significant underestimation of the amount of cellular polyP.

Finally, we examined different factors that might affect the recovery in the process of precipitating polyP with ethanol. To this end, we added to the mixture various monovalent cations, magnesium or glycogen (as carrier). As deduced from Figure 3C, none of these components had significant effect on the recovery of polyP.

### Characterizing the conditions for recombinant Ppx1-based polyP determinations

As described above, degradation of polyP by the exopolyphosphatase activity of yeast Ppx1 followed by measuring released phosphate is a common method for polyP determination. Therefore, it was considered of interest to test a number of practical aspects of this reaction suitable to be optimized. Ppx1 purified from yeast was reported to be fairly active up to 47°C 20, so we first examined the possibility to reduce the period of digestion with the enzyme by raising the incubation temperature. As shown in Figure S1A, the enzyme was highly active at 60°C but only for a relatively short period of time (20 min), followed by a sudden decline in activity, likely due to a thermal inactivation process (Figure S1B). In contrast, when incubated at 37°C, rPpx1 showed lesser, but constant activity during the experiment (60 min). As a result, incubation at 37°C resulted in a more exhaustive digestion of the polyP. However, since usually the amount of polyP to be measured is far smaller than the four µg used in this assay, it could be feasible to shorten the incubation time (and thus the duration of the assay) by raising the incubation temperature. We also tested the range of pH suitable for the use of the recombinant Ppx1 (Figure S1C). As observed, the enzyme was highly active in the pH range of 5.5 to 8.5, which fits well with the range reported for the native enzyme 20.

**Figure 4:**
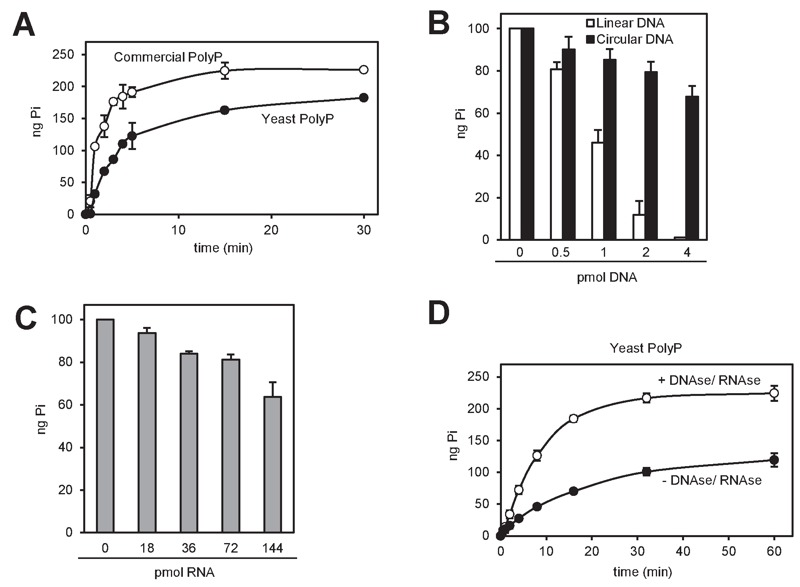
FIGURE 4: rPpx1 activity is inhibited by the presence of DNA and RNA. **(A)** Kinetic of the rPpx1 polyP digestion. rPpx1 (10 ng) was incubated with 250 ng of commercial polyP or yeast polyP, in 20 mM Tris-HCl pH 7.50 containing 5 mM magnesium acetate and 100 mM ammonium acetate at 37°C. Samples were taken at the indicated times to quantify the released Pi. Mean ± SEM from 3 independent experiments is shown. **(B)** rPpx1 activity on polyP in the presence of increasing amount of DNA. rPpx1 (10 ng) was incubated with 100 ng of commercial polyP in 20 mM Tris-HCl pH 7.50 containing 5 mM magnesium acetate and 100 mM ammonium acetate and at 37°C during 20 min with increasing concentrations of DNA (both circular and linear). The graph represents the released Pi in each condition. Mean ± SEM from 3 independent experiments is shown. **(C)** rPpx1 activity on polyP in the presence of increasing amount of RNA. Same experiment as in B, but with increasing concentrations of RNA. The graph represents the released Pi in each condition. Mean ± SEM from 3 independent experiments is shown. **(D)** Kinetics of rPpx1 polyP digestion in the presence of DNA and RNA. rPpx1 (1 ng) was incubated with 250 ng of yeast polyP previously treated or not with a DNAse/ RNAse solution in 20 mM Tris-HCl pH 7.50 containing 5 mM magnesium acetate and 100 mM ammonium acetate at 37°C. Samples were taken at the indicated times and the released Pi was quantified. Mean ± SEM from 3 independent experiments is shown.

We observed that equivalents amounts of polyP either obtained by synthesis or purified from yeast (by neutral-phenol extraction followed by ethanol precipitation) displayed different degradation kinetics, being slower that of the polymer extracted from yeast (Figure 4A). Because certain charged molecules, such as heparin 30,31 or spermidine 20, act as inhibitors of Ppx1 activity, we considered this behavior a symptom of the presence of inhibitory molecules. Ethanol precipitation is extensively used to concentrate nucleic acids, such as DNA and RNA, which are negatively charged. Given that the polyP samples used in this assay were not treated with RNAse or DNAse, and to test the possible interference of nucleic acids in the efficiency of polyP degradation, we incubated polyP with 10 ng (0.22 pmol) of rPpx1 in the presence of increasing amounts of linear or circular DNA. As showed in Figure 4B, linear DNA had a very strong inhibitory capacity, with total loss of enzymatic activity at an enzyme/DNA ratio of (20. In contrast, circular DNA was less harmful to the process. Incubation with RNA resulted in some inhibition of the enzyme, although higher amounts of ribonucleic acid were needed. As documented in Figure 4D, treatment of the ethanol precipitated material extracted from yeast with a mixture of DNAse and RNAse not only accelerates the rate of the degradation reaction but also leads to a higher value for the amount of PolyP determined. These results would suggest a functional interaction between nucleic acids (mostly linear DNA) and Ppx1. Remarkably, poly(A) polymerase, an RNA-modifying enzyme, has been found to be inhibited by polyP 32. Therefore, it must be concluded that accompanying nucleic acids can significantly interfere with the determination of polyP and that an excess of rPpx1 is recommend if it is likely the presence of nucleic acids in the assay.

### rPpx1 degradation followed by free phosphate determination offers a better linear range than DAPI quantification

An alternative to the enzymatic degradation of polyP followed by quantification of the released phosphate is the electrophoretic resolution of polyP chains and subsequent staining of the polymers with DAPI. To compare the linearity of both methods we quantified a range of polyP from 0.5 to 8 µg by both enzymatic degradation and free Pi determination and DAPI staining of electrophoretically resolved polyP samples. As shown in Figure S2A the enzymatic method was linear within the entire tested range, whereas DAPI staining, due to saturation of the signal, was linear only for amounts of polyP up to 2 µg.

### Experimental comparison of reported methods for polyP determination

Our precedent results indicate that the different steps in the determination of polyP from yeast cells can be a source of variation and could be at the origin of the large dispersions of values found in the literature (see Figure 1A). To directly test this possibility were carried out in our laboratory, for the same amount of cells, the determination of polyP using the procedures described by Neef and Kladde 33, Lichko and coworkers 23 and Hürliman *et al.* 25, in comparison with the one recently employed by our group 26. All these methods have in common the enzymatic hydrolysis of polyP with exopolyphosphatase and chemical determination of the phosphate released, but differs in the upstream methodology.

**Figure 5:**
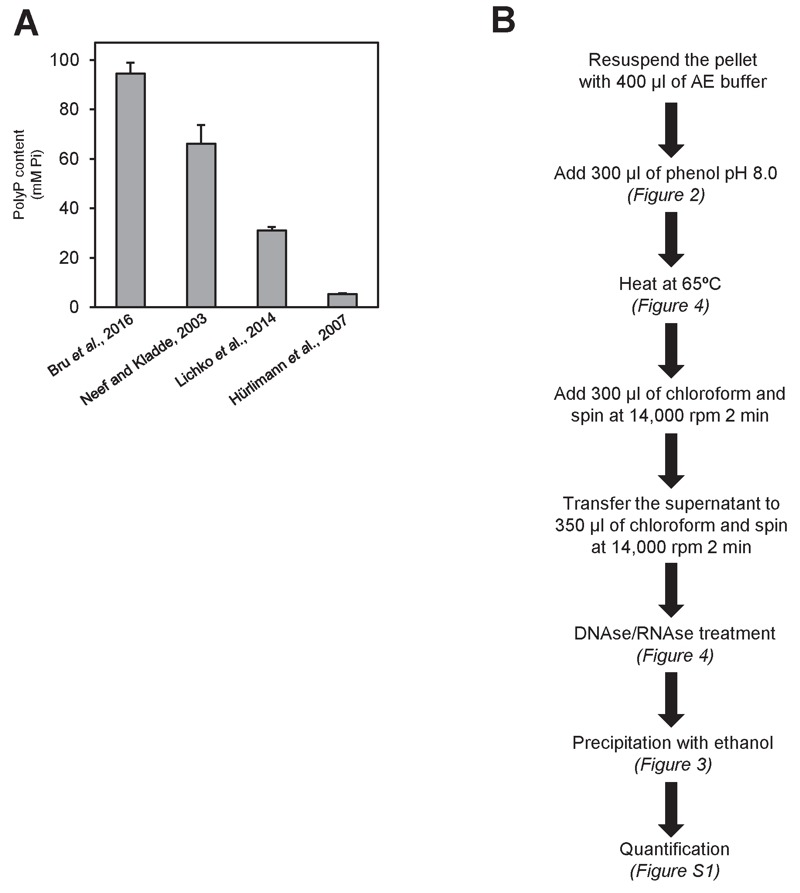
FIGURE 5: Comparison of different polyP extraction and purification methods. **(A)** Amount of polyP corresponding to 10^7^ logarithmically growing yeast cells extracted and purified with the following methods: neutral-phenol/chloroform and ethanol precipitation 26, acid-phenol/chloroform and ethanol precipitation 33, perchloric acid 23, and sulfuric acid and affinity columns 25. Mean ± SEM from 3 independent experiments is shown. **(B)** Scheme of the polyP extraction and purification protocol using neutral phenol and ethanol purification. In brackets appears the figure supporting this particular step. For details and tips see Materials and Methods.

As it can be seen in Figure 5A, the procedure described in Bru and coworkers, based in neutral phenol/chloroform extraction and ethanol precipitation after DNAse and RNAse treatment yielded an intracellular polyP concentration (expressed as Pi) of nearly 95 mM. This value is used here as reference. In contrast, the method described by Hürliman and coworkers gave the lower yield, likely because the extraction in this method is carried out by sulfuric acid treatment, which has a detrimental impact in the recovery of polyP (see Figure 2C), and because this step is followed by a Qiagen PCR column purification step, which is a second factor leading to polyP loss. Therefore, the accumulation of two detrimental steps in the entire process likely justifies the very low recovery observed for this method compared with the previous one (almost 20-fold lower). The method described by Neef and Kladde uses acid phenol and glass beads for extraction of polyP, which according to our results does not affect polyP recovery. However, these authors do not degrade nucleic acids with DNAses or RNAses prior enzymatic polyP degradation. Given the significant effect of these biomolecules on Ppx1 activity and polyP recovery (Figure 4), omission of this step could explain the somewhat lower recovery (about 30%) obtained with this procedure compared with that described in Bru *et al.* 26. Finally, the method described by Lichko and coworkers is based in the extraction of polyP with perchloric acid, which also has a detrimental effect in polyP recovery (see Figure 2C). This might explain a polyP recovery of about one third compared with the reference value.

It is important to note that the procedure utilized for polyP determination can substantially affect the conclusions extracted from a given experiment. For instance, we have observed that the marked size-specificity of the chromatography column method used in the purification step can introduce a substantial bias when comparing different physiological conditions. As an example, we recently characterized the changes in cellular polyP in yeast cells exposed to sudden alkalization of the medium by using a column chromatography purification step 34. Our results indicated a very rapid decrease, in a matter of few minutes, up to 15% of the initial polyP values. We were puzzled by such dramatic change, which was much faster than that obtained by depletion of Pi in the medium, and considered the possibility that it might be influenced by the determination procedure. Therefore, we repeated the experiment under identical conditions (using the neutral phenol extraction procedure) except that in one sample polyP was purified by affinity column and in the other the ethanol precipitation procedure was followed. As it can be observed in Figure S3, in untreated cells, utilization of the column step yields about 50% of the value obtained by the ethanol precipitation method. More importantly, according to the former method, alkalization of the medium leads to virtually complete depletion of polyP in about 2 min, whereas the later procedure yields a decrease of only 25% of the original value. Time-course electrophoretic analysis of the samples provides an explanation for such disparate results: high pH stress provokes rapid depletion of the largest polyP chains (the ones retained by the column). This result is not devoid of interest, as it might indicate that extracellular alkalization triggers the rapid activation of cellular endopolyphosphatases.

In conclusion, it is likely that most protocols described so far incur in one or more steps that negatively affect the yield of quantitated polyP and are at the basis of the dispersion of results obtained in many laboratories. Because there can be other (sometimes unavoidable) sources for variation in polyP content in yeast cells, such as the genetic background or the nature of the culture media, it is important to standardize as much as possible those factors that can be chosen at will. In this work we propose a protocol for total polyP extraction that integrates steps extracted from the literature with others shaped on the basis of the evidence presented here. This protocol is summarized in Figure 5B. Finally, our preliminary results (not shown) suggest that the protocol for polyP extraction and purification presented here can also be used for quantification of polyP from mammalian sources.

## MATERIALS AND METHODS

### Yeast strains and growing conditions

*Saccharomyces cerevisiae* BY4741 yeast strain 35 was used in all the experiments. Yeast cells were grown in 5 ml of YPD medium (1% Yeast extract, 2% Peptone, 2% Dextrose) overnight at 30°C, diluted to OD (wavelength 660 nm) = 0.4 in 10 ml of fresh YPD and let them grow to OD = 1.5. The cells were harvested by centrifugation and instantly frozen by immersion in dry ice. In the alkaline stress experiments, cells were grown and harvested as in 36. In all cases, pellets for polyP extraction were stored at -80°C.

### PolyP extraction and purification methods

#### Method 1: Neutral phenol/chloroform and ethanol precipitation

1-2*10^7^ exponentially growing cells were collected, the pellet was resuspended with 400 μl of AE buffer (50 mM sodium acetate (pH 5.3), 10 mM EDTA) at 4°C, transferred to a screw cap tube containing 300 μl phenol and 40 μl 10% SDS, mixed by inversion 4 times, vortexed 5 sec to homogenize, incubated at 65°C for 5 min and chilled for 1 min on ice. Three-hundred μl of chloroform were added, mixed by inversion 4 times, vortexed 5 sec to homogenize and centrifuged at room temperature for 2 min at 13,000 g*. *The top aqueous phase (around 450 μl) containing the polyP was transferred to a prepared 1.5 ml screw cap tube containing 350 μl chloroform (it is important not to carry over any phenol during pipetting by avoid touching the bottom phase or the white protein containing interphase), mixed by inversion 4 times, vortexed 5 sec to homogenize, centrifuged at room temperature for 2 min at 13,000 g and the aqueous phase was transferred to a new 1.5 ml microcentrifuge tube (it is important not to carry over any phenol). 2 μl of RNAse A 10 mg/ml (Sigma R6513) and 2 μl of DNAse I 10 mg/ml (Applichem, A3778.0100) were added, incubated 1 h at 37°C, transferred to a pre-cold at -20°C 1.5 ml microcentrifuge tube containing 1 ml of absolute ethanol and 40 μl of 3 M sodium acetate (pH 5.3), leaved 3 h at -20°C to precipitate polyP and centrifuged for 20 min at 13,000 g at 4°C. The supernatant was discarded by decantation, 500 μl of 70% ethanol were added, centrifuged for 5 min at 13,000 g at 4°C, the supernatant was discarded by decantation, centrifuged 1 min at 13,000 g and the last traces of ethanol were removed by pipetting. The tube was left open to dry the small translucent-white polyP pellet at room temperature for 5 min or until the pellet is completely dry. Finally, the polyP was resuspended in 50 μl of Milli-Q water. The polyP sample can be directly measured or stored at -20°C.

#### Method 2: acid phenol/chloroform and ethanol precipitation

PolyP extraction and purification was performed as described in 33. Briefly, 10^7 ^logarithmically growing yeast cells were centrifuged at 9,000 g for 1 min. Pellets were resuspended in 350 µl of LETS buffer (0.1 M LiCl, 10 mM EDTA, 10 mM Tris pH 8.0, 0.5% SDS) and 350 µl of phenol pH 4.8, lysed with the addition of 500 µl glass beads and vortexed for 15 min at 4°C. After centrifugation of the mixture at 18,000 g for 15 min, the aqueous phase was transferred to a new Eppendorf tube and subjected to chloroform extraction as above. The supernatant was precipitated by adding 1 ml of absolute ethanol followed by overnight incubation at -20°C. The pellet containing the polyP was resuspended in 50 µl of 0.1% SDS, 1 mM EDTA, 10 mM, Tris-HCl, pH 7.4.

#### Method 3: sulfuric acid extraction and affinity columns purification

PolyP extraction and purification was performed as described in 22. Briefly, 10^7^ logarithmically growing yeast cells were centrifuged at 9,000 g for 1 min. The pellets were incubated with 50 µl of 1 M sulfuric acid for 5 min at room temperature. The suspension was neutralized by adding 50 µl of 2 M NaOH and cell debris removed by centrifugation. Finally, polyP was purified using Macherey-Nagel PCR affinity purification columns, and eluted in 50 µl of MilliQ water.

#### Method 4: perchloric acid extraction and purification

PolyP extraction and purification was performed as described in 23. Briefly, 10^7^ logarithmically growing yeast cells were centrifuged at 9,000 g for 1 min. The pellet was resuspended in 250 µl of 1 M perchloric acid, lysed by adding 500 µl of glass beads, vortex for 5 min at 4°C, and centrifuged at 18,000 g for 5 min at 4°C. The suspension was neutralized by adding 150 µl of KCE solution (1 M K_2_CO_3_, 5 mM EDTA), and cooled down in ice for 2 h. Cells debris was removed by centrifugation at 18,000 g. Finally, the supernatant was transferred to a new Eppendorf tube to be quantified.

### polyP quantification

PolyP amount was determined as a measure of the inorganic phosphate produced by the complete digestion of the polyP by treatment with recombinant rPpx1 polyphosphatase protein 37; briefly, *E. coli* BL21 cells transformed with pTrcPPX1 plasmid (kindly provided by A. Kornberg) containing yeast *PPX1* were grown over night at 37°C in 50 ml of LB (Luria Bertani medium), and the culture used as inoculum to a 500 ml culture in the same LB medium containing 0.5 mM IPTG as inducer. Growth was continued for 6 h at 25°C, *E. coli* cells were harvested by centrifugation, lysed and the recombinant proteins purified using Ni-nitrilotriacetic acid agarose (Qiagen, ID:30210). The purified polyP samples to be measured were diluted in 100 µl of a solution containing 20 mM Tris-HCl (pH 7.5), 100 mM NH_4_ acetate, 5 mM Mg acetate, and 10 ng (measured by Bradford method) of rPpx1 for 1 h at 37°C. To quantify the released Pi, 86 µl of 28 mM ammonium heptamolybdate in 2.1 M sulfuric acid and 64 µl of 0.41 mM malachite green were added to the digested solution 38. The OD_595_ was measured in a Synergy HT Elisa Reader and interpolation in a standard curve was used for obtaining absolute Pi amount values.

### polyP detection by PAGE

Purified polyP was resolved electrophoretically using a 20% polyacrylamide gel (acrylamide 10:1 bisacrylamide) containing 7 M urea in TBE buffer pH 8.3, at 250 V/h for 5 h at 4°C. The dimensions of the gel were 200 mm height, 200 mm wide and 1.5 mm thick. The gel was stained by soaking it in the staining solution (25% methanol, 5% glycerol, 2 µg/ml DAPI, 50 mM Tris pH 10.5) for 30 min, and destained by soaking it in destaining solution (same as the staining solution but without DAPI) for 1 h. Finally, to visualize the polyP the gel was exposed to 254 nm UV light in Syngene G-BOX trans-illuminator.

### Assessment of rPpx1 optimal activity

The optimal temperature, and pH value for rPpx1 activity was determined by incubating 1 ng of rPpx1 and 5 µg of commercial polyP (Shiba Regenetiss, Inc.) at several temperatures and pH values respectively in 20 mM Tris-HCl pH 7.50 (when temperature was varied) containing 5 mM magnesium acetate and 100 mM ammonium acetate. In the case of the pH optimal value assessment the incubation temperature was 37°C.

The influence of DNA or RNA presence in the activity of rPpx1 was assayed using 10 ng of rPpx1 and 100 ng of commercial polyP in the same buffer as above but adding increasing amounts of DNA (circular pUC19 vector or a lineal PCR fragment of 1.7 Kb) or RNA (yeast tRNA from Sigma).

rPpx1 activity is expressed as ng of Pi released/ min/ ng of enzyme.

## SUPPLEMENTAL MATERIAL

Click here for supplemental data file.

All supplemental data for this article are also available online at http://microbialcell.com/researcharticles/improvement-of-biochemical-methods-of-polyp-quantification/.
