# Supplementary material for: Improvement of biochemical methods of polyP quantification
Source: Microb Cell. 2016 Dec 29;4(1):6–15. doi: 10.15698/mic2017.01.551 (PMC5354550; doi:10.15698/mic2017.01.551)

Supplemental figures legends

***Figure S1*. Optimal temperature and pH for rPpx1 activity.** A) Optimal temperature for rPpx1. 1 ng of rPpx1 was assayed at the noted temperatures with 5 µg of commercial polyP in 20 mM Tris-HCl pH 7.50 containing 5 mM magnesium acetate and 100 mM ammonium acetate. The graph represents the activity (ng of released Pi/ min/ ng of enzyme). Mean ± SEM from 3 independent experiments is shown. B) Kinetic of rPpx1 activity at 37 and 60ºC. 1 ng of rPpx1 and 5 µg of commercial polyP were incubated in 20 mM Tris-HCl pH 7.50 containing 5 mM magnesium acetate and 100 mM ammonium acetate. Samples were taken at different times to evaluate le amount of Pi produced. Mean ± SEM from 3 independent experiments is shown. C) Optimal pH for rPpx1. 1 ng of rPpx1 was assayed at the noted pH values with 5 µg of commercial polyP in 20 mM Tris-HCl containing 5 mM magnesium acetate and 100 mM ammonium acetate. The graph represents the activity (ng of released Pi/ min/ ng of enzyme). Mean ± SEM from 3 independent experiments is shown.

***Figure S2*. polyP quantification methods: DAPI vs malachite green. A)** Correlation between the theoretical Pi content of polyP and the detected Pi amount after rPpx1 digestion using malachite green method. rPpx1 (10 ng) was incubated with increasing concentrations of commercial polyP in 20 mM Tris-HCl pH 7.50 containing 5 mM magnesium acetate and 100 mM ammonium acetate at 37ºC during 1 h. Released Pi was quantified by malachite green method. B) Correlation between the theoretical Pi content of polyP and the detected Pi amount after rPpx1 digestion using DAPI method. PAGE and DAPI staining of the polyP samples from A at the indicated times. The quantification was using the Image Studio Lite software.

***Figure S3*. The amount of polyP obtained by ethanol precipitation is more representative of the cell polyP content than the one obtained by affinity column**. A) Analysis by PAGE and DAPI staining of the polyP obtained after precipitation or column purification of logarithmically growing yeast cells treated with the sufficient 1M KOH solution to reach pH 8.0. Samples were taken at the indicated times. polyP extraction from the yeast pellets was performed using the neutral phenol/ chloroform extraction procedure. The aqueous phase was treated with DNAse/ RNAse solution and purified with affinity columns or with ethanol precipitation. The resulting polyP fractions (precipitated, eluted and flow-through) were analyzed. B) Kinetic of the rPpx1 activity on polyP obtained from the same fractions as in panel A, polyP levels were determined from the amount of Pi released upon treatment with recombinant rPpx1. Results are from two independent experiments.

***Figure S4.* DAPI signal disappears when samples are treated with rPpx1 but not with DNase or RNase.** A) PAGE and DAPI staining analysis of polyP. polyP from a yeast pellet equivalent to 10^7 logarithmically growing yeast cells was extracted using the neutral phenol/ chloroform extraction procedure. The aqueous phase was treated with DNAse, RNAse or rPpx1 as indicated.

***Figure S5.* polyP determination in *vtc4*Δ cells**. polyP from a yeast pellet equivalent to 10^7 logarithmically growing wild-type and *vtc4*Δ yeast cells was extracted using the neutral phenol/ chloroform procedure A) PAGE and DAPI staining analysis of polyP. B) polyP was digested by treatment with rPpx1 as described in Materials and Methods, and the released Pi was measured by the malachite green method. Three independent experiments were recorded. Note that in all three experiments the *vtc4*Δ strains produced undetectable amounts of Pi and, in consequence we have drawn a line at the axis of the graph to indicate that the measures were performed.

Figure S1: Optimal temperature and pH for rPpx1 activity

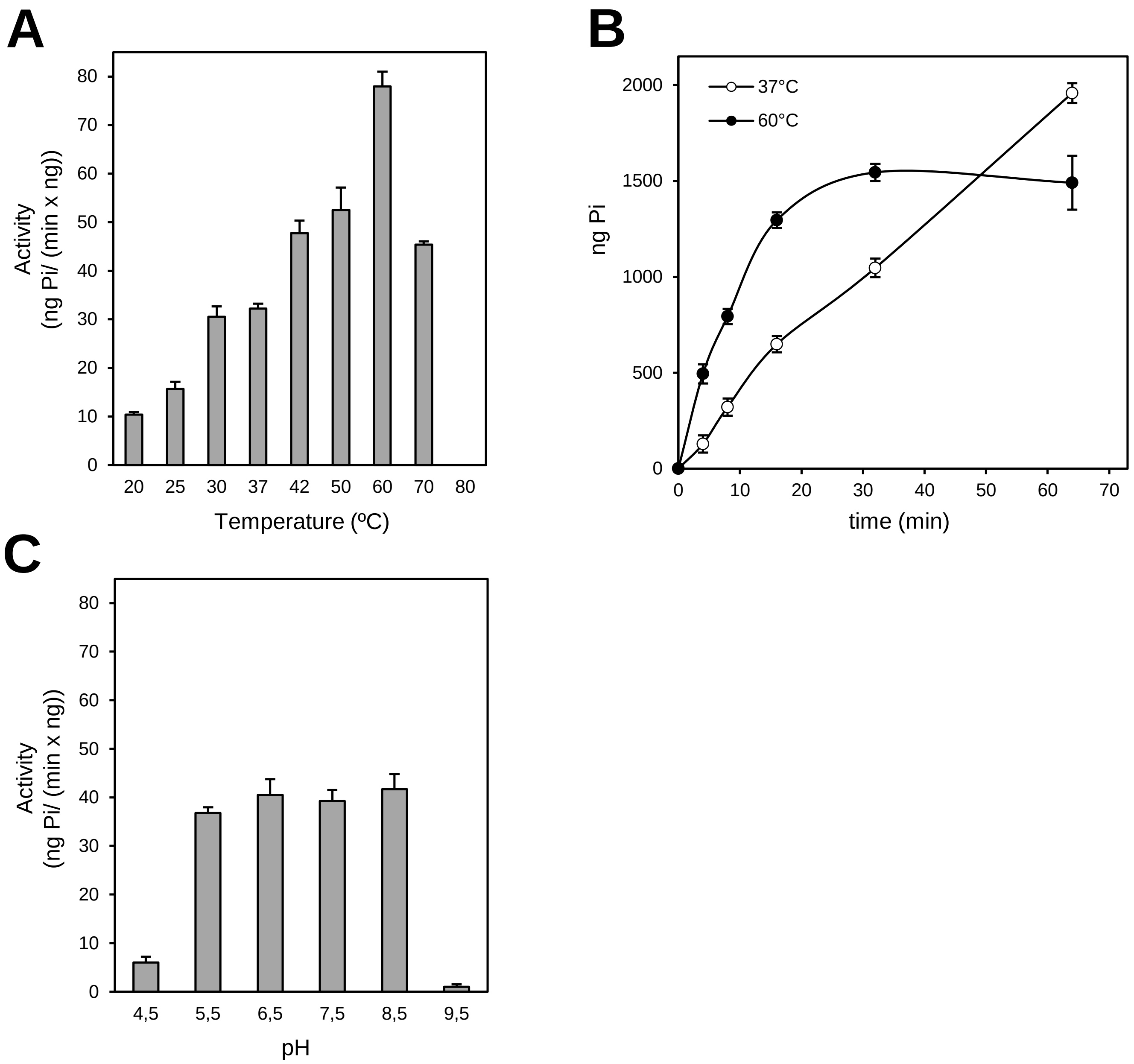

Figure S2: polyP quantification methods: DAPI vs malachite green

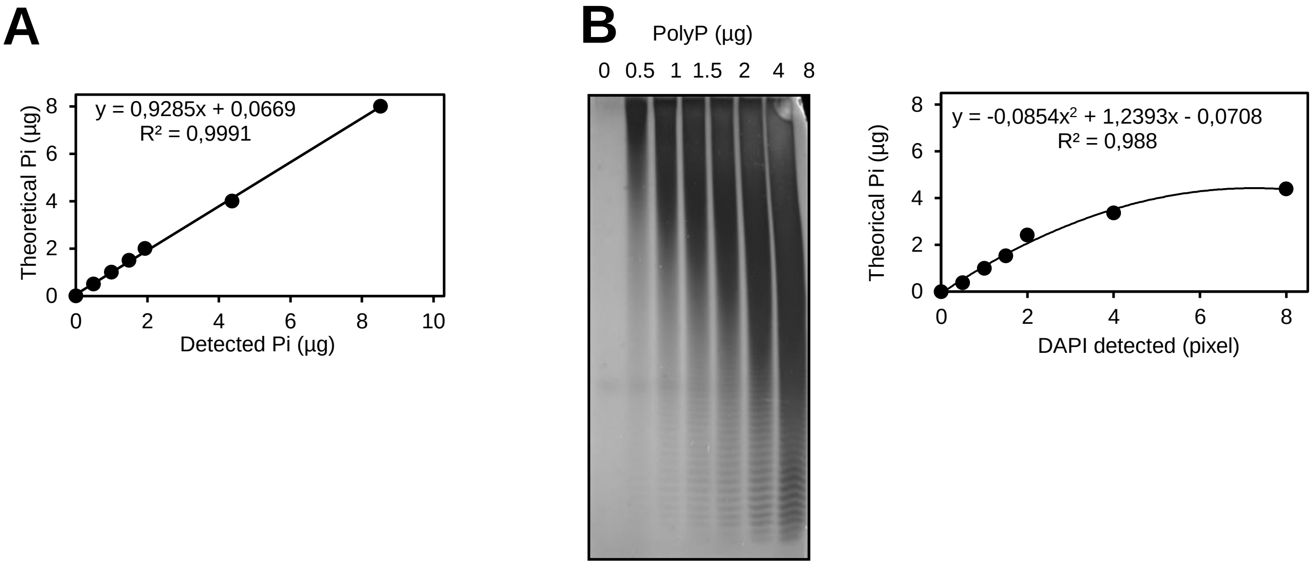

Figure S3: The amount of polyP obtained by ethanol precipitation is more representative of the cell polyP content than the one obtained by affinity column

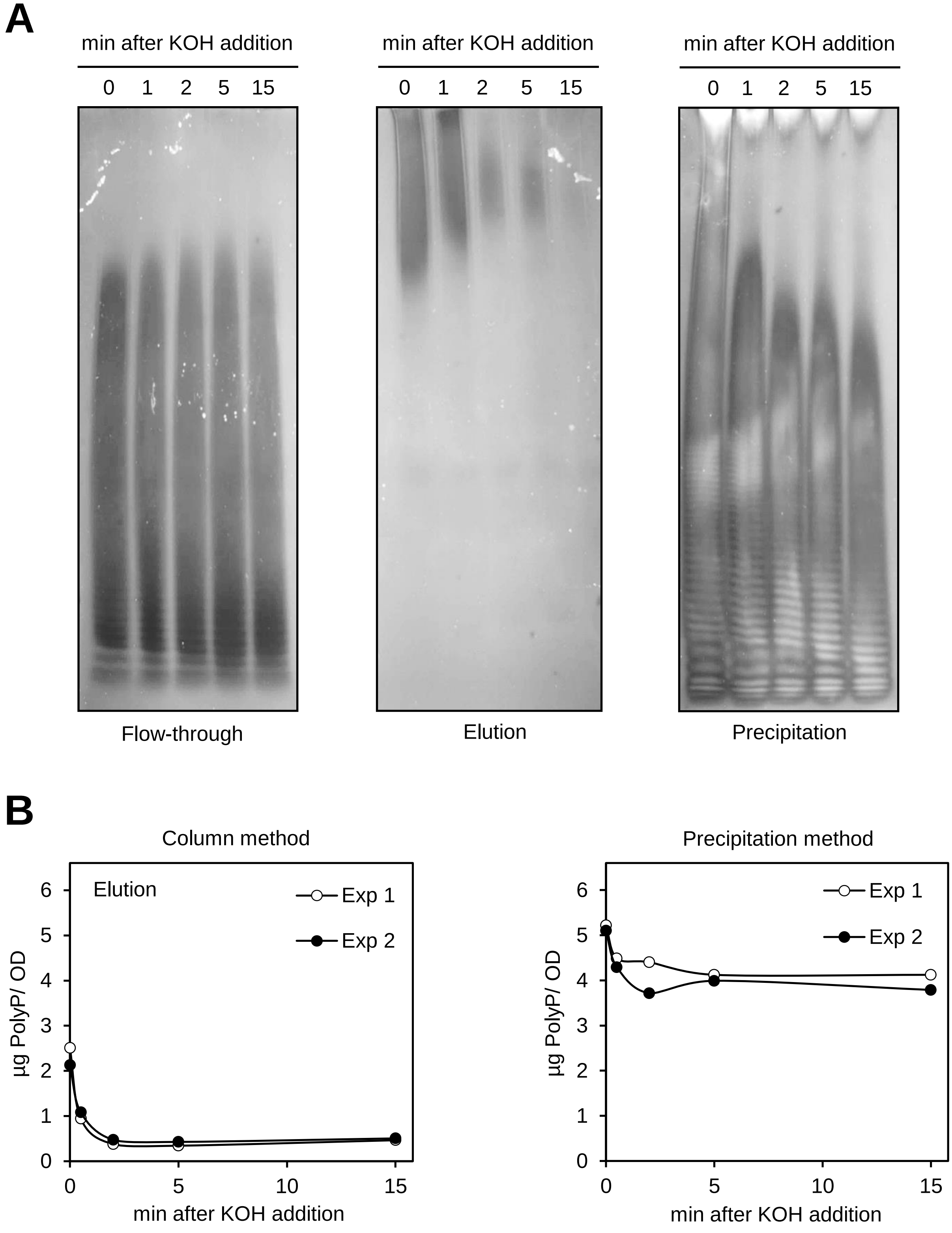

Figure S4: DAPI signal disappears when samples are treated with rPpx1 but not with DNase or RNase

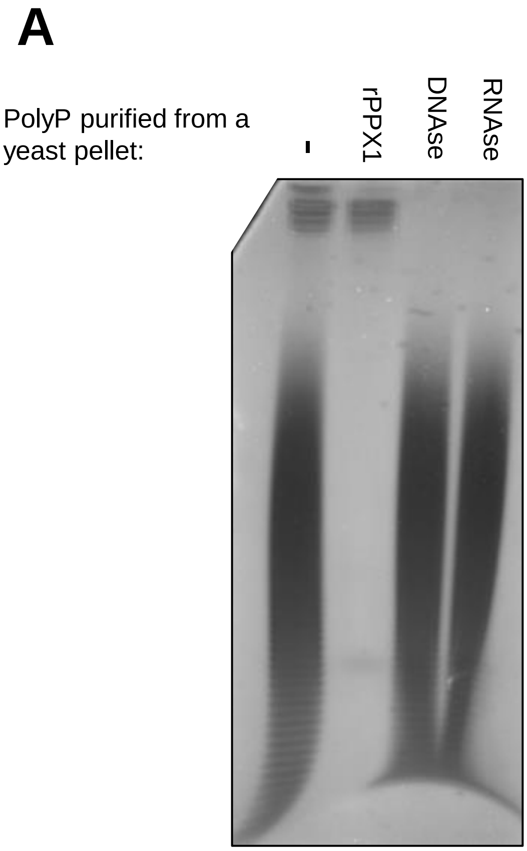

Figure S5: DAPI signal disappears when samples are treated with rPpx1 but not with DNase or RNase

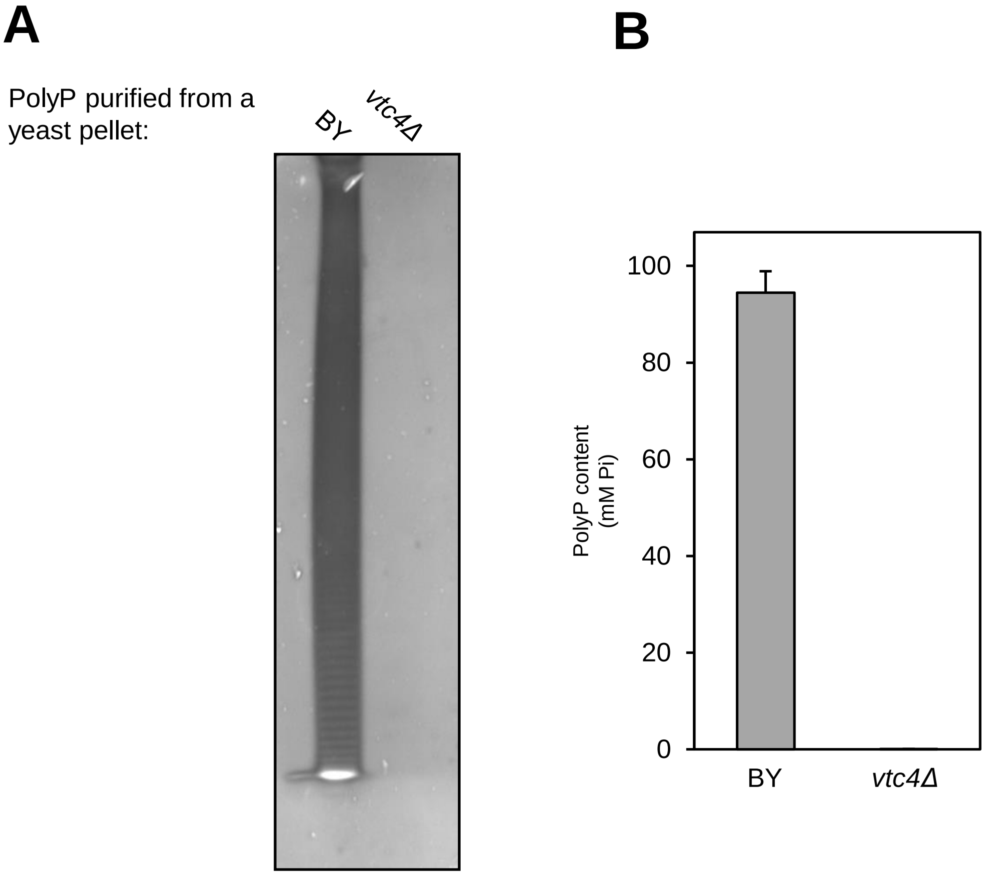

Supplement: Supplementary file 1 [file mic-04-006-s01.pdf]
